# The role of neutrophils in the pathogenesis of *Trichomonas vaginalis* infection in pregnant women: A review

**DOI:** 10.1097/MD.0000000000045063

**Published:** 2025-10-10

**Authors:** Emmanuel Ifeanyi Obeagu, Gideon Ikechukwu Okoroiwu, Nwanganga Ihuoma Ubosi, Getrude Uzoma Obeagu, Ebere Emilia Ayogu, Elham Elamin

**Affiliations:** a Department of Medical Laboratory Science, Kampala International University, Kampala, Uganda; b Department of Public Health Science, Faculty of Health Sciences, National Open University of Nigeria, Abuja, Nigeria; c Department of Nursing Science, School of Nursing Science, Kampala International University, Kampala, Uganda; d Department of Clinical Pharmacy and Pharmacy Management, Faculty of Pharmaceutical Sciences, University of Nigeria, Nsukka, Nigeria; e Department of Hematology and Immunohematology, Faculty of Medical Laboratory Sciences, University of El Imam El Mahdi, Kosti, White Nile State, Sudan.

**Keywords:** neutrophils, pathogenesis, pregnant women, *Trichomonas vaginalis*

## Abstract

*Trichomonas vaginalis (TV*) infection represents a significant public health concern, particularly among pregnant women, due to its association with adverse pregnancy outcomes such as preterm birth and low birth weight. Neutrophils, as key components of the innate immune system, play a crucial role in the host defense against *TV* infection. However, the precise role of neutrophils in the pathogenesis of *TV* infection during pregnancy remains poorly understood. Neutrophils are the first line of defense against invading pathogens, employing various mechanisms such as phagocytosis, degranulation, and the release of reactive oxygen species to eliminate microbial threats. In the context of *TV* infection, neutrophils are recruited to the site of infection in the vaginal mucosa, where they encounter and attempt to eradicate the parasite. Pregnancy imposes unique immunological challenges, characterized by dynamic alterations in the maternal immune system to accommodate the semiallogeneic fetus while maintaining protection against infections. Hormonal changes, including increased levels of progesterone and estrogen, modulate immune responses during pregnancy, potentially influencing neutrophil function and susceptibility to *TV* infection. Moreover, pregnancy-related factors such as cervical ectopy and alterations in vaginal microbiota may create a favorable environment for *TV* colonization and persistence. The implications of *TV* infection during pregnancy extend beyond maternal health, affecting neonatal outcomes and long-term child health. Preterm birth and low birth weight, often associated with *TV* infection, are significant contributors to neonatal morbidity and mortality.

## 1. Introduction

*Trichomonas vaginalis (TV*) infection represents a significant global health issue, particularly affecting pregnant women. *TV* is a protozoan parasite responsible for trichomoniasis, one of the most prevalent sexually transmitted infections (STIs) worldwide. Despite its high prevalence and potential complications, *TV* infection often goes undiagnosed and untreated, leading to adverse health outcomes, particularly in vulnerable populations such as pregnant women. The consequences of *TV* infection during pregnancy extend beyond maternal health, impacting neonatal outcomes and long-term child health. Therefore, understanding the pathogenesis of *TV* infection in pregnant women is crucial for improving maternal and neonatal health outcomes. Pregnancy imposes unique physiological and immunological changes on the maternal host to support fetal development while maintaining protection against infections. These alterations include hormonal shifts, immune modulation, and changes in the vaginal microenvironment. Hormonal changes, particularly increased levels of progesterone and estrogen, play a pivotal role in pregnancy and may influence susceptibility to infections such as *TV*. In addition, alterations in the vaginal microbiota and cervical ectopy, commonly observed during pregnancy, may create an environment conducive to *TV* colonization and persistence. Therefore, elucidating the interplay between pregnancy-associated factors and *TV* infection is essential for understanding the dynamics of *TV* pathogenesis in pregnant women.^[1–3]^

Neutrophils, as the primary responders of the innate immune system, play a crucial role in the host defense against microbial pathogens, including *TV*. Upon encountering pathogens, neutrophils are recruited to the site of infection, where they employ various effector mechanisms to eliminate the invading microorganisms. However, the role of neutrophils in the context of *TV* infection during pregnancy remains poorly understood. Emerging evidence suggests that *TV* has evolved mechanisms to evade neutrophil-mediated immune responses, leading to persistent infection and adverse pregnancy outcomes. Therefore, unraveling the complex interactions between *TV* and neutrophils is paramount for comprehensively understanding the pathogenesis of *TV* infection in pregnant women. *TV* infection during pregnancy has been associated with an increased risk of adverse pregnancy outcomes, including preterm birth, low birth weight, and perinatal complications. Preterm birth, in particular, is a leading cause of neonatal morbidity and mortality worldwide. Understanding the mechanisms underlying adverse pregnancy outcomes associated with *TV* infection is essential for developing targeted interventions to improve maternal and neonatal health. In addition, identifying biomarkers indicative of *TV* infection and its associated complications in pregnant women may facilitate early diagnosis and prompt management, thereby reducing the burden of adverse pregnancy outcomes. Despite the significant health implications of *TV* infection in pregnant women, there are several challenges in diagnosing and managing this condition. Limited access to healthcare services, stigma surrounding STIs, and lack of routine screening programs contribute to underdiagnosis and undertreatment of *TV* infection during pregnancy. Moreover, the asymptomatic nature of *TV* infection in a substantial proportion of cases further complicates detection and management efforts. Therefore, there is a critical need for enhanced awareness, improved diagnostic methods, and integrated approaches to effectively address *TV* infection in pregnant women.^[4–6]^ This review aimed to provide a comprehensive overview of the role of neutrophils in the pathogenesis of *TV* infection in pregnant women.

### 1.1. Aim

The aim of this review is to comprehensively examine the role of neutrophils in the pathogenesis of *TV* infection, specifically in pregnant women. This includes investigating the interactions between *TV*, neutrophils, and pregnancy-associated factors, with a focus on understanding the mechanisms underlying susceptibility, immune response, and adverse pregnancy outcomes associated with *TV* infection during pregnancy.

### 1.2. Rationale

The rationale for conducting this review lies in the significant impact of *TV* infection on pregnant women and their offspring, coupled with the incomplete understanding of the role played by neutrophils in the context of this infection during pregnancy. *TV* infection is a prevalent STI worldwide, and its consequences during pregnancy can be severe, including preterm birth, low birth weight, and other adverse pregnancy outcomes. Pregnant women are particularly vulnerable to the complications of *TV* infection due to physiological and immunological changes that occur during pregnancy. Neutrophils, as key components of the innate immune system, are crucial for defending the host against microbial pathogens, including *TV*. However, the specific mechanisms by which neutrophils interact with *TV* during pregnancy, and how these interactions influence maternal and neonatal health outcomes, remain poorly understood. Investigating the role of neutrophils in the pathogenesis of *TV* infection during pregnancy is essential for elucidating the underlying mechanisms of susceptibility, immune response, and adverse pregnancy outcomes associated with this infection. By conducting this review, we aim to fill existing gaps in knowledge regarding the interplay between *TV*, neutrophils, and pregnancy-related factors. Understanding these interactions may provide insights into potential targets for therapeutic interventions and diagnostic strategies tailored to *TV*-infected pregnant women. Ultimately, this review seeks to contribute to the advancement of evidence-based approaches for improving maternal and neonatal health outcomes in the context of *TV* infection during pregnancy.

## 2. Review methodology

### 2.1. Literature search strategy

A comprehensive literature search was conducted using electronic databases including PubMed, MEDLINE, Embase, and Web of Science. The search strategy included a combination of Medical Subject Headings terms and keywords related to “Trichomonas vaginalis,” “neutrophils,” “pregnancy,” and relevant synonyms. The search was limited to articles published in English language.

### 2.2. Study selection criteria

Studies were included in the review if they met the following criteria:

1. Investigated the role of neutrophils in the pathogenesis of *TV* infection in pregnant women.
2. Included human participants.
3. Provided data on outcomes related to susceptibility, immune response, or adverse pregnancy outcomes associated with *TV* infection during pregnancy.
4. Published in peer-reviewed journals.

Studies were excluded if they:

1. Were conducted exclusively in nonhuman subjects.
2. Did not focus on the role of neutrophils in *TV* infection during pregnancy.
3. Were review articles, commentaries, or editorials.

### 2.3. Quality assessment

The quality of included studies was assessed using appropriate tools such as the Newcastle–Ottawa Scale for cohort and case-control studies, and the Cochrane risk of bias tool for randomized controlled trials. Studies were evaluated based on criteria including study design, sample size, participant selection, outcome measurement, and statistical analysis.

### 2.4. Data synthesis and analysis

Data synthesis involved summarizing findings from individual studies and identifying common themes or patterns related to the role of neutrophils in *TV* infection during pregnancy. Meta-analysis was considered if appropriate, depending on the homogeneity of included studies and the availability of quantitative data.

### 2.5. TV infection in pregnancy

*TV* infection during pregnancy is a significant health concern that can have implications for both the mother and the developing fetus.^[7]^ *TV* is a protozoan parasite that causes the STI trichomoniasis.^[8]^ It is prevalent worldwide and affects individuals of all ages and genders. In pregnant women, the prevalence of *TV* infection can vary across populations but remains a notable concern due to its potential complications.^[9]^ The infection can often be asymptomatic in both pregnant and nonpregnant individuals.^[10]^ However, when symptoms occur, they might include vaginal discharge, itching, irritation, and discomfort during urination or intercourse. Diagnosis usually involves laboratory testing of vaginal swabs or urine samples to detect the presence of the parasite. Women infected with *TV* are at a higher risk of delivering prematurely, which increases the likelihood of complications for the newborn.^[11]^ Infants born to mothers with untreated trichomoniasis may have lower birth weights, which can lead to various health issues.^[12]^ *TV* infection may also increase susceptibility to other STIs and complications during pregnancy.^[13]^ Treating *TV* infection during pregnancy is crucial to reduce the risk of adverse outcomes.^[14]^ However, treatment approaches may differ due to concerns about safety during pregnancy. Some antibiotics, such as metronidazole or tinidazole, are commonly used to treat trichomoniasis, but their use during the first trimester might require careful consideration and consultation with healthcare providers. Preventive measures, such as practicing safe sex and using barrier methods like condoms, can help reduce the risk of acquiring *TV* infection.^[15]^ Regular prenatal care and screening for STIs, including trichomoniasis, are essential for pregnant individuals.^[16]^ Overall, *TV* infection during pregnancy requires attention and appropriate management to mitigate potential risks to both maternal and fetal health.^[17]^ Seeking timely medical care, adhering to treatment guidelines, and adopting preventive measures are vital aspects of managing this infection during pregnancy. Consulting healthcare professionals is crucial for proper diagnosis, treatment, and guidance concerning *TV* infection in pregnant women.^[18]^

### 2.6. Neutrophils in innate immunity

Neutrophils are a vital component of the innate immune system, serving as the body’s first line of defense against invading pathogens.^[19]^ Neutrophils are a type of white blood cell (leukocyte) that constitutes a significant portion of the body’s immune cells.^[20]^ They are highly mobile and typically the first responders to sites of infection or tissue injury. Neutrophils are characterized by their multilobed nuclei and abundant granules containing enzymes and antimicrobial substances.^[21]^ Upon encountering invading microorganisms, neutrophils are recruited to the site of infection through a process called chemotaxis.^[22]^ They exhibit phagocytic activity, engulfing and destroying pathogens through a process known as phagocytosis. In addition, neutrophils release antimicrobial molecules, such as reactive oxygen species and enzymes stored in their granules, to kill and degrade pathogens.^[23]^ Neutrophils can also release extracellular structures called neutrophil extracellular traps (NETs) as a defense mechanism.^[24]^ NETs are composed of DNA, histones, and antimicrobial proteins, forming a web-like structure that traps and neutralizes pathogens, preventing their spread.^[25]^ Neutrophils play a significant role in the inflammatory response.^[26]^ Upon activation, they release cytokines and chemokines, signaling molecules that recruit other immune cells to the site of infection, amplifying the immune response. Neutrophils have a relatively short lifespan, typically circulating in the bloodstream for a few hours before migrating to tissues or undergoing apoptosis (programmed cell death).^[27]^ They are continuously produced in the bone marrow and regulated by various factors, including cytokines and growth factors. Neutrophil dysfunction or abnormalities can lead to increased susceptibility to infections.^[28]^ Conditions such as neutropenia (a low count of neutrophils) or impaired neutrophil function can result in compromised immune responses and recurrent infections. While primarily associated with innate immunity, recent research suggests that neutrophils also interact with components of the adaptive immune system. They can modulate T-cell responses, influence the activation of other immune cells, and participate in shaping the overall immune response to pathogens. Neutrophils serve as essential effectors of the innate immune system, playing a critical role in recognizing, engulfing, and destroying pathogens as part of the body’s defense against infections.^[29]^ Their multifaceted functions contribute significantly to maintaining immune homeostasis and protecting the body from microbial threats.

### 2.7. Neutrophils and TV infection

Neutrophils, as key components of the innate immune system, play a crucial role in the body’s response to infections, including *TV*.^[30]^ When *TV* infects the urogenital tract, neutrophils are among the first immune cells recruited to the site of infection.^[31]^ The presence of the parasite triggers a local inflammatory response, leading to the migration of neutrophils to the infected area through a process called chemotaxis. Neutrophils employ phagocytosis – a process involving engulfment of the pathogen – to attempt to eliminate *TV*.^[32]^ These cells attempt to engulf and destroy the parasite using their granules containing antimicrobial substances and enzymes. However, the effectiveness of neutrophils in completely clearing *TV* from the urogenital tract may vary. *TV* can modulate neutrophil functions, potentially affecting their ability to eradicate the parasite.^[33]^ Studies suggest that the parasite might interfere with neutrophil migration, impair phagocytosis, or influence the release of inflammatory mediators by neutrophils.^[34–36]^ This modulation might contribute to the persistence of the infection in some cases. Neutrophils also release NETs in response to *TV* infection.^[37]^ NETs are structures composed of DNA, histones, and antimicrobial proteins that trap and neutralize pathogens. However, whether NETs play a significant role in combating *TV* remains an area of ongoing research. The interaction between neutrophils and *TV* infection can influence the course and severity of the infection. In some instances, a robust neutrophil response might help limit the spread of the parasite. However, the ability of *TV* to evade or modulate neutrophil responses may contribute to the persistence of the infection in the urogenital tract, leading to chronic or recurrent cases.^[38]^

### 2.8. Immunomodulatory effects of TV on neutrophils

*TV*, the protozoan parasite responsible for trichomoniasis, exhibits various immunomodulatory effects that can impact the functions and responses of neutrophils, a critical component of the innate immune system.^[39]^ Studies suggest that *TV* can interfere with the normal functioning of neutrophils.^[40,41]^ The parasite might affect the ability of neutrophils to perform essential functions such as chemotaxis (movement toward sites of infection), phagocytosis (engulfing and destroying pathogens), and the release of antimicrobial substances. This impairment could diminish the effectiveness of neutrophils in combating the parasite. *TV* might interfere with the migration and activation of neutrophils, potentially disrupting their response to the infection.^[42]^ By modulating signaling pathways or altering the expression of certain molecules involved in neutrophil activation, the parasite can hinder the ability of neutrophils to reach and effectively combat *TV* in the urogenital tract. The parasite has developed mechanisms to evade or resist neutrophil-mediated killing. It might possess surface molecules or proteins that help it evade recognition by neutrophils or counteract the antimicrobial activities of these immune cells. These strategies could contribute to the persistence of *TV* in the host despite the presence of neutrophil responses. *TV* can trigger inflammatory responses in the urogenital tract.^[43]^ While neutrophils are involved in the inflammatory process as part of the immune response, the parasite-induced inflammation might not always lead to effective elimination of the pathogen. Instead, it could create an environment conducive to the parasite’s survival or allow for chronic infection. The parasite might influence the production and release of cytokines by neutrophils. This modulation of cytokine responses could contribute to the overall inflammatory milieu in the urogenital tract, affecting the balance of immune responses and potentially influencing the course of *TV* infection. Understanding the mechanisms by which *TV* modulates neutrophil functions and responses is crucial for elucidating the strategies employed by the parasite to evade host immune defenses.^[44]^

### 2.9. Neutrophil-derived inflammation and pregnancy outcomes

Neutrophils, as integral components of the immune system, play a significant role not only in defending against infections but also in contributing to inflammation, which can have implications for pregnancy outcomes.^[45]^ Inflammation is a natural response of the immune system to infections, tissue injury, or stress. During pregnancy, a delicate balance of immune responses is necessary to support fetal development while protecting the mother from infections. However, excessive or dysregulated inflammation can potentially impact pregnancy outcomes.^[46–50]^ Neutrophils are key contributors to the inflammatory process.^[51]^ Upon activation, they release cytokines, chemokines, and other inflammatory mediators. While inflammation is essential for combating infections, an exaggerated or prolonged inflammatory response can lead to tissue damage and adverse effects. Excessive or uncontrolled inflammation, including that derived from neutrophil activity, has been associated with various adverse pregnancy outcomes.^[52]^ Chronic inflammation during pregnancy may contribute to complications such as preterm birth, pre-eclampsia, intrauterine growth restriction, and fetal developmental abnormalities. In particular, elevated levels of inflammatory markers, including those associated with neutrophil activation, have been linked to an increased risk of preterm birth and low birth weight. Chronic inflammatory conditions or infections during pregnancy may trigger an inflammatory cascade that could potentially lead to premature delivery and adverse fetal growth. The mechanisms linking neutrophil-derived inflammation to adverse pregnancy outcomes are complex and multifaceted.^[53]^ Excessive inflammation might induce placental dysfunction, disrupt fetal development, or contribute to uterine contractions and cervical changes leading to preterm labor. Monitoring inflammatory markers and managing conditions associated with excessive inflammation during pregnancy could be important for reducing the likelihood of adverse outcomes. Research focusing on modulating inflammatory responses, including those involving neutrophils, may offer potential therapeutic avenues to manage inflammatory conditions during pregnancy. Identifying ways to regulate and balance the immune response without compromising fetal development is an area of ongoing investigation. While inflammation, including those involving neutrophils, is a fundamental aspect of the body’s defense mechanisms, its dysregulation during pregnancy may contribute to adverse outcomes.^[54]^

### 2.10. Clinical implications

Developing sensitive and specific diagnostic methods for detecting *TV* infection in pregnant women is crucial.^[55]^ Improved diagnostics can facilitate early identification and timely treatment, reducing the risk of adverse pregnancy outcomes. Studying the safety and efficacy of antimicrobial treatments for *TV* in pregnant women is essential. Understanding the impact of treatment regimens on both maternal and fetal health is critical to ensure appropriate management. Integrating routine screening for *TV* infection into prenatal care protocols can aid in identifying and managing infections early in pregnancy.^[16]^ This can potentially minimize the risk of complications and improve pregnancy outcomes. Exploring interventions that modulate the immune response, particularly neutrophil functions, against *TV* could be beneficial.^[56]^ Strategies to enhance the host immune response or mitigate immune evasion mechanisms employed by the parasite may be promising avenues for future research.

### 2.11. Future perspectives

#### 2.11.1. Current research advances

Recent research has made significant strides in elucidating the role of neutrophils in the pathogenesis of *TV* infection in pregnant women. Studies have provided valuable insights into the interactions between *TV*, neutrophils, and pregnancy-associated factors, shedding light on mechanisms underlying susceptibility, immune response, and adverse pregnancy outcomes. Furthermore, advances in diagnostic techniques and therapeutic interventions have improved our ability to detect and manage *TV* infection during pregnancy.^[57]^

#### 2.11.2. Lacunae in available literature

Despite recent research advances, several lacunae persist in the available literature, indicating areas for further investigation:

1. *Mechanistic understanding*: While existing studies have highlighted the involvement of neutrophils in *TV* infection during pregnancy, the precise mechanisms by which neutrophils interact with *TV* and contribute to adverse pregnancy outcomes remain incompletely understood. Future research should focus on unraveling the molecular pathways involved in neutrophil recruitment, activation, and effector functions during *TV* infection in pregnant women.
2. *Impact of microbiota*: The role of the vaginal microbiota in modulating neutrophil responses to *TV* infection during pregnancy is an area requiring further exploration. Understanding how alterations in the vaginal microbiota influence neutrophil function and *TV* pathogenesis may provide novel insights into preventive and therapeutic strategies.
3. *Immunomodulatory factors*: Pregnancy-related hormonal changes and immune modulation play a significant role in shaping the immune response to *TV* infection. However, the specific effects of hormones and immune mediators on neutrophil function in the context of *TV* infection during pregnancy are not fully elucidated. Investigating the immunomodulatory effects of pregnancy-associated factors on neutrophil responses may uncover new targets for intervention.
4. *Long-term outcomes*: While the immediate impact of *TV* infection during pregnancy on adverse pregnancy outcomes is well-documented, the long-term consequences for maternal and child health remain understudied. Longitudinal studies are needed to assess the persistence of *TV* infection, its effects on maternal reproductive health, and the developmental outcomes of offspring exposed to *TV* in utero.
5. *Therapeutic interventions:* The development of effective therapeutic interventions for *TV* infection during pregnancy is hampered by limited understanding of the immunopathogenesis of the disease. Future research should focus on identifying novel drug targets and evaluating the efficacy and safety of existing treatment regimens in pregnant women.

### 2.12. Directions for further research

1. *Mechanistic studies*: Conduct mechanistic studies to elucidate the molecular interactions between *TV* and neutrophils during pregnancy, focusing on pathways involved in neutrophil recruitment, activation, and effector functions.^[58]^
2. *Microbiota–neutrophil interactions*: Investigate the impact of vaginal microbiota on neutrophil responses to *TV* infection during pregnancy, exploring potential mechanisms of microbiota-mediated modulation of immune responses.
3. *Hormonal and immunomodulatory effects*: Explore the effects of pregnancy-related hormones and immune mediators on neutrophil function in the context of *TV* infection, using in vitro and animal models to dissect underlying mechanisms.
4. *Longitudinal studies*: Conduct longitudinal studies to assess the long-term effects of *TV* infection during pregnancy on maternal reproductive health and offspring development, incorporating follow-up assessments beyond the perinatal period.
5. *Therapeutic trials*: Initiate clinical trials to evaluate the efficacy and safety of novel therapeutic agents for *TV* infection in pregnant women, considering factors such as drug penetration, fetal safety, and potential for drug resistance.

## 3. Conclusion

*TV* infection poses a significant health concern for pregnant women, potentially leading to adverse outcomes for both the mother and the developing fetus. Neutrophils, as pivotal components of the innate immune system, play a crucial role in responding to infections, including *TV*, and shaping the immune environment during pregnancy. The interaction between *TV* and neutrophils is complex, involving various mechanisms that impact the host’s immune response. Neutrophils are recruited to the site of infection, attempting to eliminate the parasite through phagocytosis and the release of antimicrobial substances. However, the parasite’s ability to modulate neutrophil functions can lead to immune evasion, potentially contributing to the persistence of the infection and affecting pregnancy outcomes.

Understanding the immunomodulatory effects of *TV* on neutrophils is essential for advancing clinical care and developing targeted interventions. Addressing diagnostic strategies, treatment considerations, and the integration of screening protocols into prenatal care can aid in early detection and management of *TV* infections during pregnancy. Future research directions should focus on elucidating the specific mechanisms by which *TV* evades neutrophil responses, exploring immunomodulatory strategies that enhance the host immune response, and assessing the long-term implications of the infection on maternal and fetal health outcomes. A comprehensive understanding of the interplay between neutrophils and *TV* infection in pregnant women is crucial for improving clinical care, guiding therapeutic interventions, and ultimately safeguarding maternal and fetal well-being.

## Author contributions

**Conceptualization:** Emmanuel Ifeanyi Obeagu.

**Methodology:** Emmanuel Ifeanyi Obeagu, Getrude Uzoma Obeagu.

**Supervision:** Emmanuel Ifeanyi Obeagu.

**Validation:** Emmanuel Ifeanyi Obeagu.

**Visualization:** Emmanuel Ifeanyi Obeagu.

**Writing – original draft:** Emmanuel Ifeanyi Obeagu, Gideon Ikechukwu Okoroiwu, Nwanganga Ihuoma Ubosi, Getrude Uzoma Obeagu, Ebere Emilia Ayogu, Elham Elamin.

**Writing – review and editing:** Emmanuel Ifeanyi Obeagu, Gideon Ikechukwu Okoroiwu, Nwanganga Ihuoma Ubosi, Getrude Uzoma Obeagu, Ebere Emilia Ayogu, Elham Elamin.
